# Lack of metabolism in (*R*)-ketamine’s antidepressant actions in a chronic social defeat stress model

**DOI:** 10.1038/s41598-018-22449-9

**Published:** 2018-03-05

**Authors:** Kai Zhang, Yuko Fujita, Kenji Hashimoto

**Affiliations:** 1 Division of Clinical Neuroscience, Chiba University Center for Forensic Mental Health, Chiba, Japan; 2 Wuxi Mental Health Center, Nanjing Medical University, Wuxi, China

## Abstract

Since the metabolism of (*R,S*)-ketamine to (2*R*,6*R*)-hydroxynorketamine (HNK) is reported to be essential for ketamine’s antidepressant effects, there is an increasing debate about antidepressant effects of (2*R*,6*R*)-HNK. Using pharmacokinetic and behavioral techniques, we investigated whether intracerebroventricular (i.c.v.) infusion of (*R*)-ketamine or (2*R*,6*R*)-HNK show antidepressant effects in a chronic social defeat stress (CSDS) model of depression. Low levels of (2*R*,6*R*)-HNK in the brain after i.c.v. infusion of (*R*)-ketamine were detected, although brain levels of (2*R*,6*R*)-HNK were markedly lower than those after i.c.v. infusion of (2*R*,6*R*)-HNK. Furthermore, high levels of (2*R*,6*R*)-HNK in the blood and liver after i.c.v. infusion of (*R*)-ketamine or (2*R*,6*R*)-HNK were detected. A single i.c.v. infusion of (*R*)-ketamine showed rapid and long-lasting (7 days) antidepressant effects in a CSDS model. In contrast, i.c.v. infusion of (2*R*,6*R*)-HNK did not show any antidepressant effect in the same model, although brain concentration of (2*R*,6*R*)-HNK was higher than after i.c.v. infusion of (*R*)-ketamine. This study suggest that (*R*)-ketamine in the periphery after washout from the brain is metabolized to (2*R*,6*R*)-HNK in the liver, and subsequently, (2*R*,6*R*)-HNK enters into brain tissues. Furthermore, it is unlikely that (2*R*,6*R*)-HNK is essential for the antidepressant actions of (*R*)-ketamine in a CSDS model.

## Introduction

Depression is a common, severe, and chronic psychiatric disease. Although current antidepressants have been used to treat depression, their beneficial effects are limited. Berman *et al*. reported that a subanesthetic dose of ketamine, an NMDAR (*N*-methyl-D-aspartate receptor) antagonist, elicits rapid and sustained antidepressant effects in depressed patients^1^. Subsequent clinical studies exert rapid and sustained antidepressant effects in treatment-resistant patients with major depression or bipolar disorder^2–8^. However, the precise mechanisms underlying ketamine’s actions remain unclear^9–14^.

**Figure 1:**
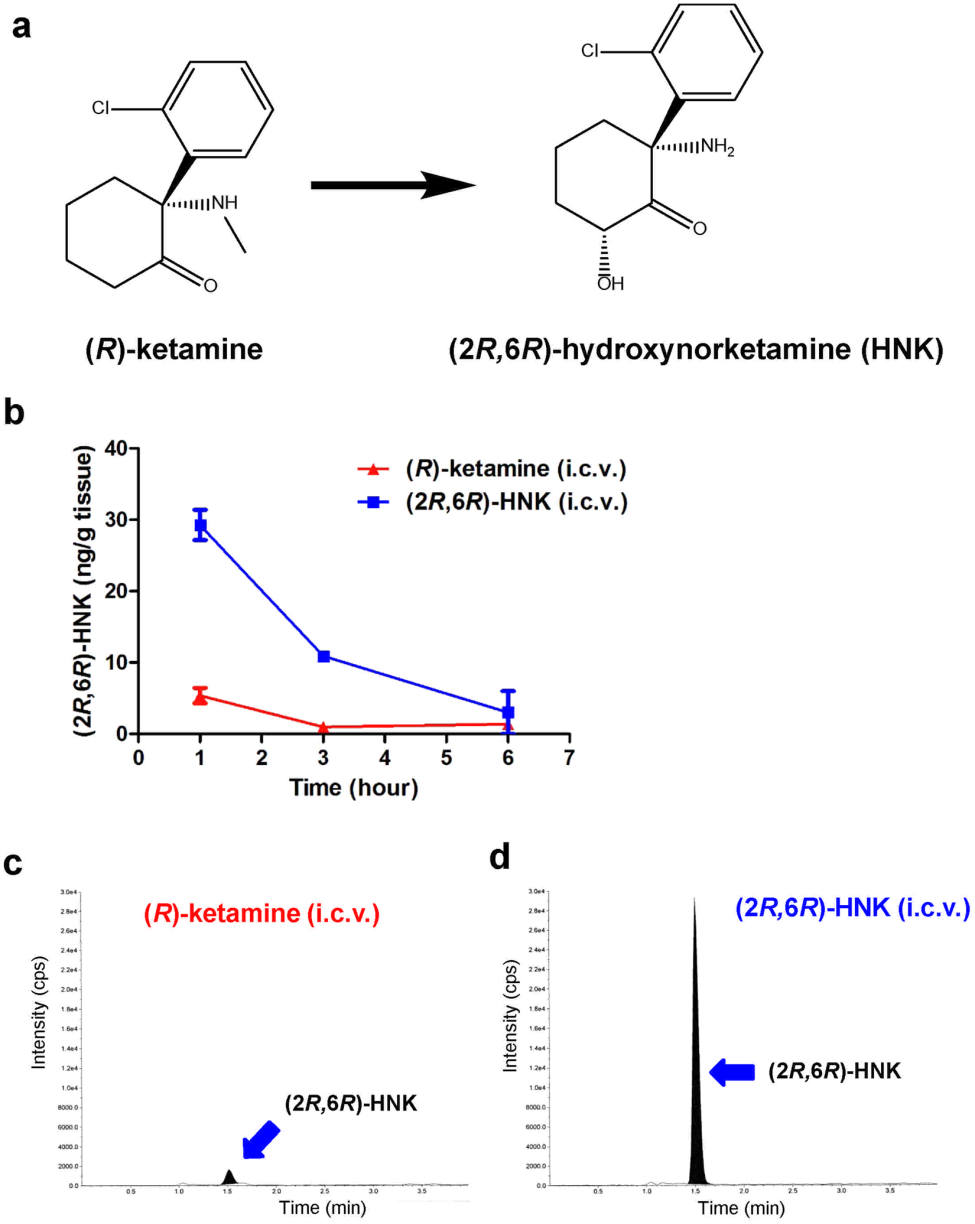
Metabolism of (*R*)-ketamine to (2*R*,6*R*)-HNK, and determination of (2*R*,6*R*)-HNK in the brain after i.c.v. infusion of (*R*)-ketamine or (2*R*,6*R*)-HNK. (**a**) (*R*)-ketamine is rapidly metabolized in the liver by microsomal cytochrome P450 enzymes into (2*R*,6*R*)-hydroxynorketamine (HNK). (**b**) Brain concentrations of (2*R*,6*R*)-HNK in the brain 1, 3 and 6 hours after a single i.c.v. infusion of (*R*)-ketamine or (2*R*,6*R*)-HNK. Data are shown as mean ± SEM. [n = 3: n = 2 for (2*R*,6*R*)-HNK (6 hr)]. (**c**) Typical chromatogram of the brain sample 1 hour after i.c.v. infusion of (*R*)-ketamine. (**d**) Typical chromatogram of the brain sample 1 hour after i.c.v. infusion of (2*R*,6*R*)-HNK.

In 2016, Zanos *et al*. reported that the metabolism of (*R,S*)-ketamine to (2*R*,6*R*)-hydroxynorketamine (HNK) is essential for the antidepressant effects of ketamine in rodents, in an NMDAR inhibition-independent manner^15^. However, the recent study showed a critical role of NMDAR inhibition-mediated signaling of (2*R*,6*R*)-HNK^16,17^. It is well known that ketamine is rapidly metabolized in the liver by microsomal cytochrome P450 enzymes into norketamine (through *N*-demethylation) and finally into HNK^15,18–23^ (Fig. 1a). Several groups have reported that (*R*)-ketamine (Ki = 1.40 μM for NMDAR) showed greater potency and longer-lasting antidepressant effects than (*S*)-ketamine (Ki = 0.30 μM for NMDAR) in animal models of depression^15,24–29^, suggesting that NMDAR inhibition and other unknown mechanisms may play a key role in the ketamine’s antidepressant actions^12–14^. Interestingly, Zanos *et al*. claimed that (2*R*,6*R*)-HNK (>10 μM for NMDAR), a final metabolite from (*R*)-ketamine, plays a key role in the ketamine’s antidepressant actions^15^. However, we did not replicate antidepressant effects of (2*R*,6*R*)-HNK in a chronic social defeat stress (CSDS) model^27^ and a learned helplessness (LH) model^30^. There is now an increasing debate about the antidepressant actions of (2*R*,6*R*)-HNK in rodents^12–14,27,30–35^.

To exclude the metabolism of ketamine to HNK in the liver, this study was undertaken to examine whether intracerebroventricular (i.c.v.) infusion of (*R*)-ketamine or its metabolite (2*R*,6*R*)-HNK shows antidepressant effects in a CSDS model of depression. First, using a liquid chromatography tandem mass spectrometry (LC-MS/MS), we determined the concentration of (2*R*,6*R*)-HNK in the brain, blood and liver after i.c.v. injection of (*R*)-ketamine and (2*R*,6*R*)-HNK. Second, we examined whether i.c.v. infusion of (*R*)-ketamine and (2*R*,6*R*)-HNK shows antidepressant effects in a CSDS model.

## Results

### Concentration of (2*R*,6*R*)-HNK after i.c.v. infusion of (*R*)-ketamine or (2*R*,6*R*)-HNK

Using LC-MS/MS, we first measured the concentration of (2*R*,6*R*)-HNK in the brain samples after i.c.v. infusion of (*R*)-ketamine or (2*R*,6*R*)-HNK. Unexpectedly, lower levels of (2*R*,6*R*)-HNK in the brain were detected 1, 3, and 6 hours after i.c.v. infusion of (*R*)-ketamine, which gradually declined (Fig. 1b,c). In contrast, higher levels of (2*R*,6*R*)-HNK in the brain were detected after i.c.v. infusion of (2*R*,6*R*)-HNK, which gradually declined (Fig. 1b,d). In particular, the tissue concentration of (2*R*,6*R*)-HNK in the brain 1 hour after i.c.v. infusion of (2*R*,6*R*)-HNK was markedly higher than that of (2*R*,6*R*)-HNK 1 hour after i.c.v. infusion of (*R*)-ketamine.

**Figure 2:**
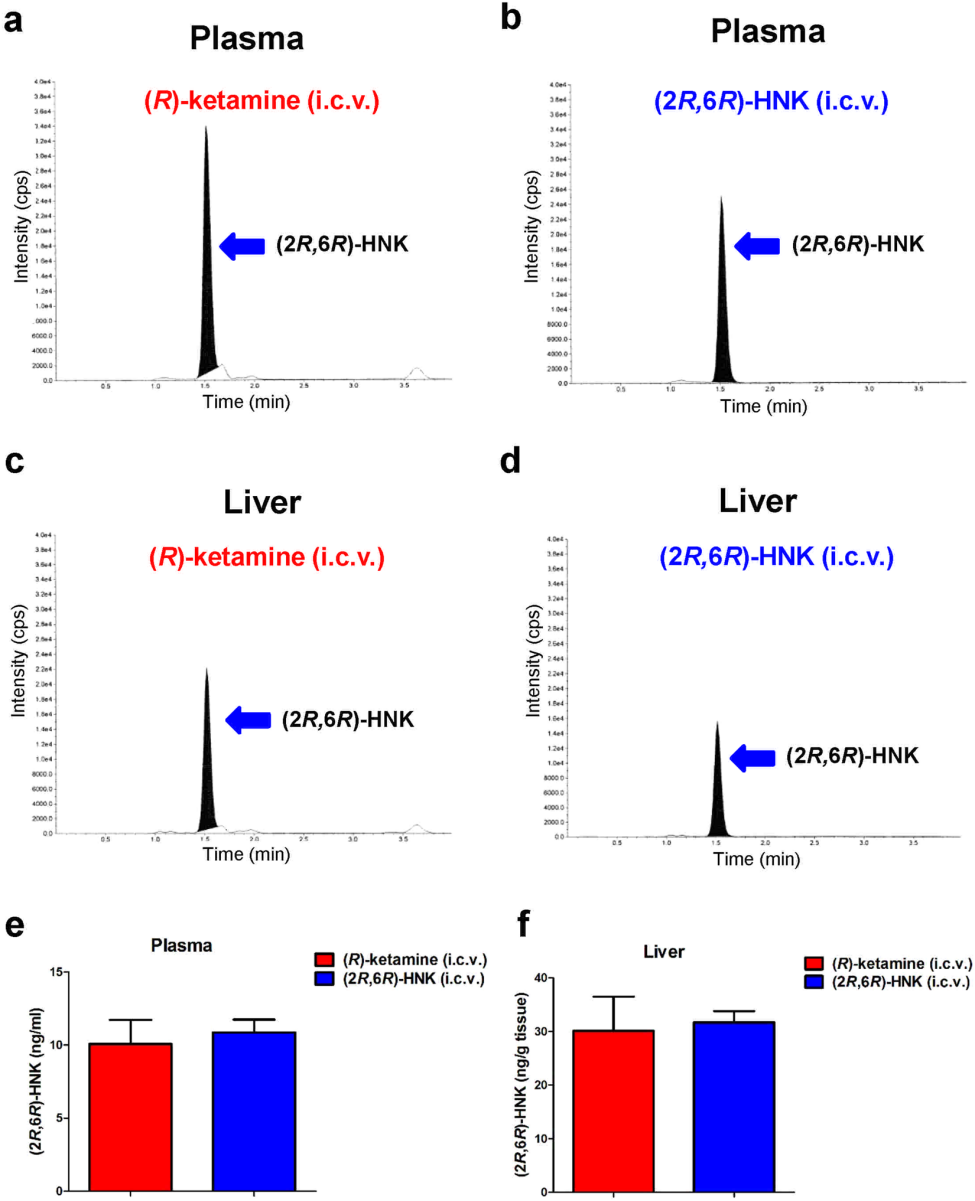
Determination of (2*R*,6*R*)-HNK in the plasma and liver after i.c.v. infusion of (*R*)-ketamine or (2*R*,6*R*)-HNK. (**a**) Typical chromatogram of (2*R*,6*R*)-HNK in the blood (or plasma) 1 hour after a single i.c.v. infusion of (*R*)-ketamine. (**b**) Typical chromatogram of (2*R*,6*R*)-HNK in the blood (or plasma) 1 hour after a single i.c.v. infusion of (2*R*,6*R*)-HNK. (**c**) Typical chromatogram of (2*R*,6*R*)-HNK in the liver 1 hour after a single i.c.v. infusion of (*R*)-ketamine. (**d**) Typical chromatogram of (2*R*,6*R*)-HNK in the liver 1 hour after a single i.c.v. infusion of (2*R*,6*R*)-HNK. (**e)** Concentration of (2*R*,6*R*)-HNK in the plasma 1 hour after a single i.c.v. infusion of (*R*)-ketamine or (2*R*,6*R*)-HNK. (**f**) Concentration of (2*R*,6*R*)-HNK in the liver 1 hour after a single i.c.v. infusion of (*R*)-ketamine or (2*R*,6*R*)-HNK. Data are shown as mean ± SEM. [n = 3 for (*R*)-ketamine: n = 2 for (2*R*,6*R*)-HNK].

Next, we examined whether (2*R*,6*R*)-HNK was detected in the blood and liver 1 hour after i.c.v. infusion of (*R*)-ketamine or (2*R*,6*R*)-HNK. High levels of (2*R*,6*R*)-HNK were detected in the blood and liver 1 hour after i.c.v. infusion of (*R*)-ketamine (Fig. 2a,c). Furthermore, high levels of (2*R*,6*R*)-HNK were detected in the blood and liver 1 hour after i.c.v. infusion of (2*R*,6*R*)-HNK (Fig. 2b,d), indicating the existence of (2*R*,6*R*)-HNK in the periphery after washout from the brain. Interestingly, the concentration of (2*R*,6*R*)-HNK in the plasma and liver after i.c.v. infusion of (*R*)-ketamine or (2*R*,6*R*)-HNK was the same (Fig. 2e,f).

### Antidepressant effects of i.c.v. infusion of (*R*)-ketamine or (2*R*,6*R*)-HNK

**Figure 3:**
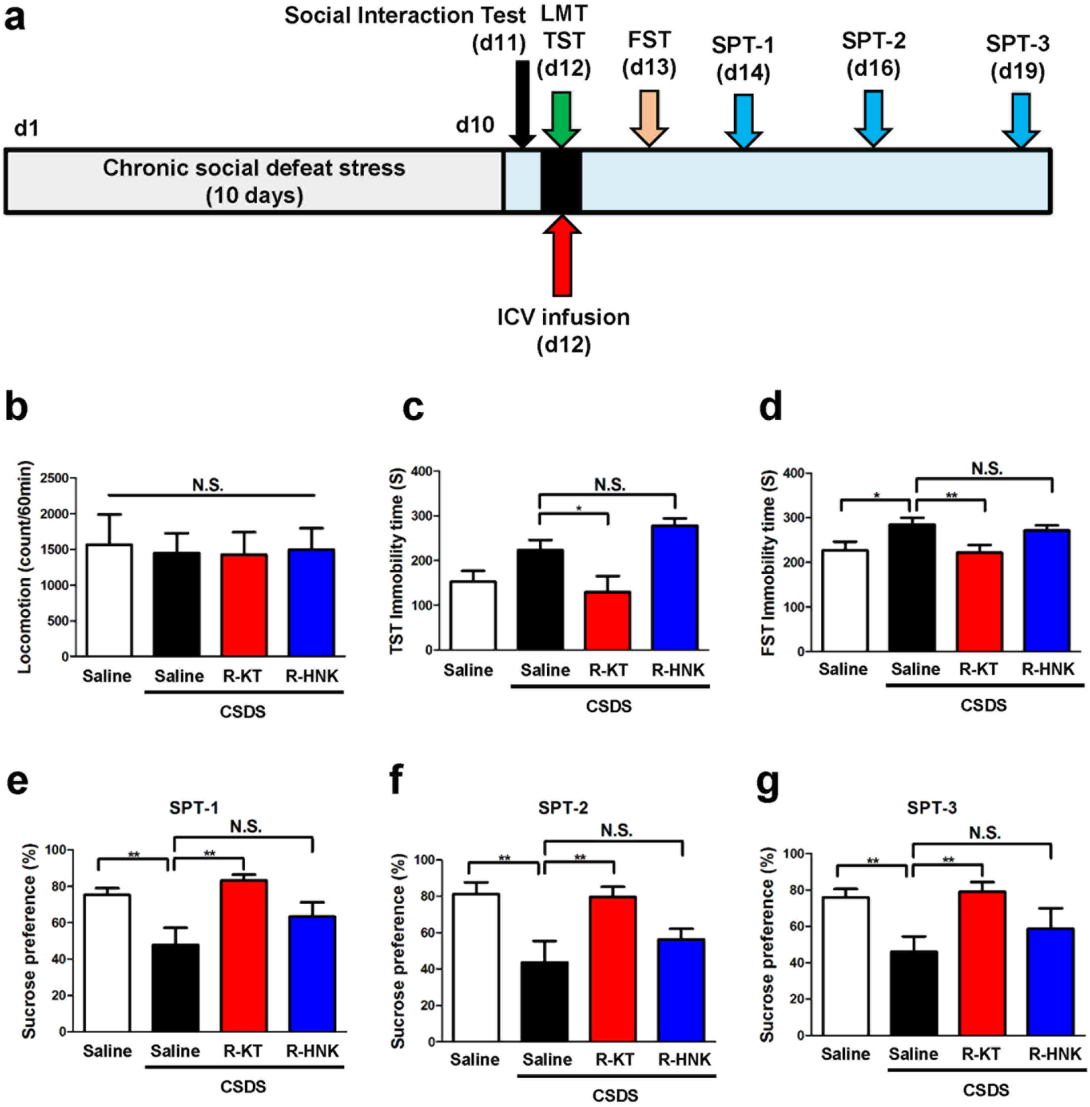
Antidepressant actions of i.c.v. infusion of (*R*)-ketamine, but not (2*R*,6*R*)-HNK, in a CSDS model. (**a**) CSDS was performed from day 1 to day 10, and the social interaction test (SIT) was performed on day 11. Saline (2 μl), (*R*)-ketamine (10 mg/ml, 2 μl) or (2*R*,6*R*)-HNK (10 mg/ml, 2 μl) was administered i.c.v. to CSDS susceptible mice on day 12. Locomotion (LMT) and tail suspension test (TST) were performed 2 and 4 hours after a single infusion, respectively. Forced swimming test (FST) was performed 24 hours after a single infusion. One % sucrose preference test (SPT) was performed 2, 4 and 7 days after a single infusion. (**b**) Locomotion (LMT) (one-way ANOVA, F_3,32_ = 0.04, P = 0.99). (**c**) TST (one-way ANOVA, F_3,32_ = 6.90, P < 0.001). (**d**) FST (one-way ANOVA, F_3,32_ = 4.11, P = 0.02). (**e**) SPT-1 at d14 (one-way ANOVA, F_3,32_ = 5.50, P < 0.001). (**f**) SPT-2 at d16 (one-way ANOVA, F_3,32_ = 5.75, P < 0.001). (**g**) SPT-3 at d19 (one-way ANOVA, F_3,32_ = 4.35, P = 0.01). Data are shown as mean ± SEM. (n = 8). *P < 0.05, **P < 0.01 compared to saline-treated group of CSDS susceptible mice. R-KT: (*R*)-ketamine. R-HNK: (2*R*,6*R*)-HNK. N.S.: not significant.

Next, we examined whether i.c.v. infusion of (*R*)-ketamine or (2*R*,6*R*)-HNK showed antidepressant effects in a CSDS model (Fig. 3a). There were no differences in locomotion among the four groups (Fig. 3b). In the TST, i.c.v. infusion of (*R*)-ketamine, but not (2*R*,6*R*)-HNK, significantly reduced the increased immobility time of susceptible mice after the induction of CSDS (Fig. 3c). In the FST, i.c.v. infusion of (*R*)-ketamine, but not (2*R*,6*R*)-HNK, significantly reduced the increased immobility time of CSDS-susceptible mice 1 day after a single infusion (Fig. 3d). In the SPT, i.c.v. infusion of (*R*)-ketamine, but not (2*R*,6*R*)-HNK, showed anti-anhedonia effects 2, 4, or 7 days after a single infusion (Fig. 3e–g). These data suggest that, unlike that of (*R*)-ketamine, i.c.v. infusion of (2*R*,6*R*)-HNK does not elicit rapid and long-lasting antidepressant effects in a CSDS model.

## Discussion

In the present study, we found evidence of (2*R*,6*R*)-HNK in the brain after i.c.v. infusion of (*R*)-ketamine, although brain concentration of (2*R*,6*R*)-HNK was lower than those after i.c.v. infusion of (2*R*,6*R*)-HNK. Furthermore, we detected high concentration of (2*R*,6*R*)-HNK in the blood and liver after i.c.v. infusion of (*R*)-ketamine. These data suggest that (*R*)-ketamine in the periphery after washout from the brain is metabolized to (2*R*,6*R*)-HNK in the liver, and subsequently, (2*R*,6*R*)-HNK enters into brain tissues. In addition, we also found high concentration of (2*R*,6*R*)-HNK in the blood and liver after i.c.v. infusion of (2*R*,6*R*)-HNK, indicating the rapid washout into periphery from the brain. Nonetheless, the concentration of (2*R*,6*R*)-HNK in the brain after i.c.v. infusion of (*R*)-ketamine was lower than that after i.c.v. infusion of (2*R*,6*R*)-HNK.

In this study, we found that i.c.v. infusion of (*R*)-ketamine showed rapid and long-lasting antidepressant effects in a CSDS model, consistent with previous reports using intraperitoneal administration^25,27–29,36,37^. In contrast, i.c.v. infusion of (2*R*,6*R*)-HNK did not show any antidepressant effects in a CSDS model, although the concentrations of (2*R*,6*R*)-HNK in the brain were higher than those after i.c.v. infusion of (*R*)-ketamine. Furthermore, we reported that intraperitoneal administration of (*R*)-ketamine, but not (2*R*,6*R*)-HNK, shows rapid and long-lasting antidepressant effects in a CSDS model and an inflammation-induced model^27^. In a rat LH model, we recently reported that (2*R*,6*R*)-HNK (20 or 40 mg/kg, 24 h and 5 days) did not elicit any antidepressant effects in LH rats, although (*R*)-ketamine (20 mg/kg) showed sustained (24 h) and long-lasting (5 days) antidepressant effects in the same model^30^. In addition, we reported that a single bilateral infusion of (*R*)-ketamine into the infralimbic (IL) region of the medial prefrontal cortex (mPFC), CA3, and dentate gyrus of the hippocampus shows long-lasting (5 days) antidepressant effects in a rat LH model^38^. A previous study also demonstrated that microinfusion of (*R,S*)-ketamine into IL of mPFC produces antidepressant-like effects in control unstressed rats^39^. Collectively, it is likely that (*R*)-ketamine itself in the brain can exert antidepressant effects in rodents with depression-like phenotype and that (2*R*,6*R*)-HNK in the brain does not have antidepressant effects in rodents with depression-like phenotype.

Recently, Pharm *et al*. reported that similar to (*R,S*)-ketamine (10 mg/kg, 24 h)-induced antidepressant-like effects, (2*R*,6*R*)-HNK (10 mg/kg, 24 h) increased the swimming duration (in the forced swimming test) and extracellular 5-hydroxytryptamine level in the medial prefrontal cortex (mPFC) of control naive mice^40^. Furthermore, a single bilateral injection of (*R,S*)-ketamine (2 nmol, 24 h) or (2*R*,6*R*)-HNK (2 nmol, 24 h) into mPFC significantly increased the swimming duration of control naive mice. This study showed that (*R,S*)-ketamine and (2*R*,6*R*)-HNK have an equal intensity of antidepressant-like effects in control naïve mice, and that (*R,S*)-ketamine itself has antidepressant-like effects because (*R,S*)-ketamine is not metabolized to (2*R*,6*R*)-HNK in this brain region. These findings^38–40^ obtained using intra-cortical infusion support the idea that (2*R*,6*R*)-HNK is not the primary mediator of the antidepressant-like effects of (*R,S*)-ketamine,^15^ since (2*R*,6*R*)-HNK is not prepared in the brain regions.

In addition, Zanos *et al*. reported that the highly potent NMDAR antagonist (+)-MK-801 (Ki = 30.5 nM for NMDAR) failed to elicit antidepressant effects lasting 24 hours in FST^15^. Unlike (*R,S*)-ketamine, (+)-MK-801 did not reverse social avoidance induced by CSDS, indicating a lack of antidepressant activity of (+)-MK-801 in a CSDS model. From these findings, Zanos *et al*. concluded that there are NMDAR inhibition-independent mechanisms underlying ketamine’s antidepressant effects^15^. In contrast, many previous reports showed that (+)-MK-801 had antidepressant-like effects in control naïve rodents^41–46^. In addition, we reported that (+)-MK-801 induces rapid antidepressant effects in a CSDS model, although this response is not long-lasting^47^. Taking these findings together, it is possible that NMDAR inhibition and other unknown mechanisms may play a role in the long-lasting (7 days) antidepressant actions of ketamine, although NMDAR inhibition may play a role in a rapid antidepressant effect^47^.

Recently, Yao *et al*.^48^ reported that a single intraperitoneal injection of (*R,S*)-ketamine (10 mg/kg, 1 day) impaired long-term potentiation (LTP) in the nucleus accumbens (NAc) of control mice but had no effects on the basic properties of glutamatergic transmission in this region. This loss of LTP in the NAc was maintained for 7 days, consistent with the long-lasting antidepressant actions of (*R,S*)-ketamine. Furthermore, a single injection of (2*R*,6*R*)-HNK (10 mg/kg, 1 day) also impaired LTP in the NAc of control mice. Interestingly, (*R,S*)-ketamine (10 mg/kg) and its enantiomers (*R*)- and (*S*)-ketamine (10 mg/kg) significantly attenuated reduced dendritic spine density, brain-derived neurotrophic factor (BDNF) and its receptor TrkB signaling, and GluA1/PSD-95 expression in the medial prefrontal cortex (mPFC) and hippocampus (CA3 and DG) of mice with a depression-like phenotype, but did not alter the corresponding elevations in NAc^25,28,49^. In the rat LH model, we also reported that a single bilateral infusion of (*R*)-ketamine into the infralimbic region of mPFC, CA3, and DG improved depression-like symptoms, whereas a single bilateral infusion of (*R*)-ketamine into the NAc did not induce antidepressant effects^38^. These findings suggest that (*R,S*)-ketamine and its enantiomers exert antidepressant effects by normalizing BDNF−TrkB signaling and synaptogenesis in the mPFC and hippocampus, but not NAc. Taken together, it is unlikely that NAc plays a direct role in the antidepressant actions of (*R,S*)-ketamine and its two enantiomers.

In conclusion, the present study demonstrates that i.c.v. infusion of (*R*)-ketamine, but not its final metabolite (2*R*,6*R*)-HNK, could elicit a rapid and long-lasting antidepressant effect in a CSDS model, although low tissue concentrations of (2*R*,6*R*)-HNK were detected in the brain after i.c.v. infusion of (*R*)-ketamine. The present data argue against the claim made by a paper that stated that (2*R*,6*R*)-HNK is essential for the antidepressant actions of (*R,S*)-ketamine [or (*R*)-ketamine]^15^. Finally, we propose that (*R*)-ketamine, through NMDAR inhibition and subsequent unidentified mechanisms (for instance, synaptogenesis via BDNF–TrkB signaling)^25,50–52^, promotes rapid and longer-lasting antidepressant actions.

## **M**ethods and Materials

### Animals

Male adult C57BL/6 mice, aged 8 weeks (body weight 20–25 g, Japan SLC, Inc., Hamamatsu, Japan) and male adult CD1 (ICR) mice, aged 13–15 weeks (body weight > 40 g, Japan SLC, Inc., Hamamatsu, Japan) were used. Animals were housed under controlled temperatures and 12 hour light/dark cycles (lights on between 07:00–19:00 h), with ad libitum food (CE-2; CLEA Japan, Inc., Tokyo, Japan) and water. The study was approved by the Chiba University Institutional Animal Care and Use Committee (Permission number: 29–406). This study was carried out in strict accordance with the recommendations in the Guide for the Care and Use of Laboratory Animals of the National Institutes of Health, USA. All efforts were made to minimize suffering.

### Materials

(*R*)-ketamine hydrochloride was prepared as previously reported^24^. The purity of (*R*)-ketamine was determined by a high-performance liquid chromatography with a chiral column as previously reported^24^. (2*R*,6*R*)-HNK hydrochloride was purchased from Sigma-Aldrich Co, Ltd (St Louis, MO, USA).

### Determination of (2*R*,6*R*)-HNK in the brain, blood, and liver after i.c.v. infusion of (*R*)-ketamine or (2*R*,6*R*)-HNK

Determination of (2*R*,6*R*)-HNK in the mouse samples was performed using the previous reports^15,23,26^ with a slight modification.

Experiment 1: The mice were anesthetized deeply with 5% isoflurane, and Neuros Syringes (702 N 25 μl SYR, Hamilton Company, Reno, NV, USA) were placed into the lateral ventricles (+0.02 AP, +1.0 ML, −1.5 DV)^53^. Subsequently, (*R*)-ketamine (10 mg/ml, 2 μl, i.c.v.) or (2*R*,6*R*)-HNK (10 mg/ml, 2 μl, i.c.v.) was administered to the mice. The mice were anesthetized deeply with 5% isoflurane, and they were sacrificed by decapitation. Then the brain samples excluding cerebellum were collected at each sampling time point (1, 3, and 6 hour). The excised brain was rinsed in ice-cold saline and then homogenized with 4 volumes of distilled water to prepare a brain homogenate specimen.

Experiment 2: The mice were anesthetized deeply with 5% isoflurane, and Neuros Syringes (702 N 25 μl SYR, Hamilton Company, Reno, NV, USA) were placed into the lateral ventricles (+0.02 AP, +1.0 ML, −1.5 DV)^53^. Subsequently, (*R*)-ketamine (10 mg/ml, 2 μl, i.c.v.) or (2*R*,6*R*)-HNK (10 mg/ml, 2 μl, i.c.v.) was administered into the mice. The mice were anesthetized deeply with 5% isoflurane 1 hour after injection, and blood was placed into a tube containing ethylenediamine-*N*,*N*,*N*’,*N*’-tetraacetic acid dipotassium salt dihydrate (EDTA·2 K) as an anticoagulant. Blood samples were immediately centrifuged (3,000 × g, 3 min) to prepare plasma samples. Liver samples were also collected. The excised liver was rinsed in ice-cold saline and then homogenized with 4 volumes of distilled water to prepare a liver homogenate specimen. These biological specimens were stored at −80 °C until bioanalysis.

Determination of (2*R*,6*R*)-HNK in the mouse samples was performed at Sumika Chemical Analysis Service, Ltd (Osaka, Japan). A 50-µl aliquot of the plasma or brain (or liver) homogenate specimen was mixed with 25 µl of 1 mM ammonium hydrogen carbonate/acetonitrile (7:3, v/v), 20 µl of acetonitrile/methanol (9:1, v/v) containing ^2^H_4_-norketamine (Sigma-Aldrich Co, Ltd, St. Louis, MO, USA) as an internal standard (I.S.) and 100 µl of 1 mM ammonium hydrogen carbonate. And t-butyl methyl ether, 2 mL, was added and vortex-mixed for 1 minute. After centrifugation at 3,000 rpm for 5 minutes, the organic layer was transferred to another empty glass tube. The solvent was evaporated to dryness under a stream of nitrogen gas at 25 °C. The residue was dissolved in 100 μl of 1 mM ammonium hydrogen carbonate/acetonitrile (7:3, v/v) by vortex-mixing for 30 seconds and sonicating for 1 minute. The solution was centrifuged at 3,000 rpm for 5 minutes. A 5-µl aliquot of the supernatant resulting from the pretreatment was subjected to an enantioselective liquid chromatography tandem mass spectrometry (LC-MS/MS) assay. The LC-MS/MS system was constructed using a Shimadzu LC-20A high-performance liquid chromatography system (Shimadzu, Tokyo, Japan) and API5000 tandem mass spectrometer (AB SCIEX, Foster City, CA, USA). The MS/MS data were acquired and processed using Analyst version 1.6.1 software (AB SCIEX, Foster City, CA). Chromatographic separation was performed at 25 °C on a CHIRALPAK AS-3R analytical column (4.6 mm i.d. × 100 mm, 3 µm particles, Daicel Corporation, Tokyo, Japan) using 1 mM ammonium hydrogencarbonate/acetonitrile (54:46, v/v) as a mobile phase at a flow rate of 1.0 ml/min. The selected reaction monitoring transition of (2*R*,6*R*)-HNK was m/z 240.5 → m/z 125.0, and the I.S. was m/z 228.1 → m/z 129.1. The lower limit of quantification (LLOQ) in the brain and liver was 0.5 ng/g tissue. The LLOQ in plasma was 0.1 ng/ml.

### Chronic social defeat stress (CSDS) model

CSDS was performed as previously reported^25,27–29,36,37,48,54,55^. The C57BL/6 mice were exposed to a different CD1 aggressor mouse for 10 min/day for 10 days. After the social defeat session, the resident CD1 mouse and the intruder C57BL/6 mouse were housed in one half of the cage separated by a perforated Plexiglas divider to allow visual, olfactory, and auditory contact for the remainder of the 24-hour period. All mice were housed individually 24 hour after the last social defeat stress session. On day 11, a social interaction test (SIT) was performed to divide susceptible group and resilient group to CSDS. The test was accomplished by placing mice in an interaction test box (42 × 42 cm) with an empty wire-mesh cage (10 × 4.5 cm) located at one end. The movement of the mice was tracked for 2.5 min, followed by 2.5 min in the presence of an unfamiliar aggressor CD1 mouse confined in the wire-mesh cage. The duration of the subject’s presence in the “interaction zone” (defined as the 8-cm-wide area surrounding the wiremesh cage) was recorded by a stopwatch. The interaction ratio was calculated as time spent in an interaction zone with an aggressor / time spent in an interaction zone without an aggressor. The cutoff for an interaction ratio was set as 1. Mice with scores < 1 were defined as “susceptible” to social defeat stress, and mice with scores ≥ 1 were defined as “resilient”. Only susceptible mice were used in the behavioral experiments. Control C57BL/6 mice not exposed CSDS were housed in the home cage before the behavioral tests.

### Behavioral tests in a CSDS model

The CSDS susceptible mice and control mice were anesthetized deeply with 5% isoflurane, and Neuros Syringes (702 N 25 μl SYR, Hamilton Company, Reno, NV, USA) were placed into the lateral ventricles (+0.02 AP, +1.0 ML, −1.5 DV)^53^. Subsequently, vehicle (saline, 2 μl, i.c.v.), (*R*)-ketamine (10 mg/ml, 2 μl, i.c.v.) or (2*R*,6*R*)-HNK (10 mg/ml, 2 μl, i.c.v.) was administered to CSDS susceptible mice. Vehicle (saline, 2 μl, i.c.v.) was also administered to control mice. Behavioral tests were performed as reported previously^25,27–29,36,37,54,55^.

### Locomotion

Locomotion was performed 2 hour after i.c.v. infusion. The locomotor activity was determined using an animal movement analysis system (SCANET MV-40, MELQUEST Co., Ltd., Toyama, Japan). Mice were placed in experimental cages (length × width × height: 560 × 560 × 330 mm). The cumulative locomotor activity was recorded for 60 minutes. Cages were cleaned between testing session.

### Tail suspension test (TST)

TST was performed 4 hour after i.c.v. infusion. A small piece of adhesive tape placed approximately 2 cm from the tip of the mouse tail. A single hole was punched in the tape and mice were hung individually, on a hook. The TST immobility time was recorded for 10 minutes. Mice were considered immobile only when they hung passively and completely motionless.

### Forced swimming test (FST)

FST was performed 24 hours after i.c.v. infusion. The FST was performed using an automated forced-swim system SCANET MV-40 (MELQUEST Co., Ltd., Toyama, Japan). The mice were placed individually in a cylinder (diameter: 23 cm; height: 31 cm) containing 15 cm of water (23 ± 1 °C). FST immobility time from activity time as (total) – (active) time for 6 minutes was calculated.

### Sucrose preference test (SPT)

SPT was performed 2, 4, 7 days after i.c.v. infusion. Mice were exposed to water and 1% sucrose solution for 48 h, followed by 4 hours of water and food deprivation. The two identical bottles containing water and 1% sucrose were weighed before and at the end of this period (1 hour). The sucrose preference was calculated as a percentage of sucrose solution consumption to the total liquid consumption.

### Statistical analysis

The data show as the mean ± standard error of the mean (S.E.M.). Analysis was performed using PASW Statistics 20 (formerly SPSS Statistics; SPSS). Comparisons between groups were performed using the one-way analysis of variance (ANOVA), followed by *post-hoc* Tukey test. The P-values of less than 0.05 were considered statistically significant.
