# Author Correction: Lack of metabolism in (*R*)-ketamine’s antidepressant actions in a chronic social defeat stress model

**DOI:** 10.1038/s41598-018-28860-6

**Published:** 2018-07-12

**Authors:** Kai Zhang, Yuko Fujita, Kenji Hashimoto

**Affiliations:** 1 Division of Clinical Neuroscience, Chiba University Center for Forensic Mental Health, Chiba, Japan; 2 Wuxi Mental Health Center, Nanjing Medical University, Wuxi, China

Correction to: *Scientific Reports* 10.1038/s41598-018-22449-9, published online 05 March 2018

This Article contains errors in References 18 and 40 which are incorrectly given as:

18. Yanagisawa, Y. *et al*. Involvement of CYP2B6 in *N*-demethylation of ketamine in human liver microsomes. *Drug Metab*. *Dispos*. **29**, 887–890 (2001).

40. Pharm, T. H. *et al*. Common neurotransmission recruited in (*R*,*S*)-ketamine and (2*R*,6*R*)-hydroxynorketamine-induced sustained antidepressant effects. *Biol*. *Psychiatry* 10.1016/j.biopsych.2017.10.020 (in press).

The correct references are listed below as References 1 and 2.
